# Could a simple antenatal package combining micronutritional supplementation with presumptive treatment of infection prevent maternal deaths in sub-Saharan Africa?

**DOI:** 10.1186/1471-2393-7-6

**Published:** 2007-05-23

**Authors:** Simon M Collin, Rebecca F Baggaley, Rudiger Pittrof, Veronique Filippi

**Affiliations:** 1 Department of Social Medicine, University of Bristol, Canynge Hall, Bristol, BS8 2PR, UK; 2 Department of Epidemiology & Population Health, London School of Hygiene and Tropical Medicine, Keppel Street, London, WC1E 7HT, UK; 3 Enfield Town Clinic, Wenlock House, 33 Eaton Road, Enfield, EN1 1NJ, UK

## Abstract

**Background:**

Reducing maternal mortality is a key goal of international development. Our objective was to determine the potential impact on maternal mortality across sub-Saharan Africa of a combination of dietary supplementation and presumptive treatment of infection during pregnancy. Our aim was to demonstrate the importance of antenatal interventions in the fight against maternal mortality, and to stimulate debate about the design of an effective antenatal care package which could be delivered at the lowest level of the antenatal health system or at community level.

**Methods:**

We collated evidence for the effectiveness of antenatal interventions from systematic reviews and controlled trials, and we selected interventions which have demonstrated potential to prevent maternal deaths. We used a model-based analysis to estimate the total reduction in maternal mortality in sub-Saharan Africa which could be achieved by combining these interventions into a single package, based on a WHO systematic review of causes of maternal deaths.

**Results:**

Severe hypertensive disorders, puerperal sepsis and anemia are causes of maternal deaths which could be prevented to some extent by prophylactic measures during pregnancy. A package of pills comprising calcium and iron supplements and appropriate anti-microbial and anti-malarial drugs could reduce maternal mortality in sub-Saharan Africa by 8% (range <1% to 20%). This estimate is based on Cochrane Review estimates for the effectiveness of daily calcium supplements in reducing the risk of death/serious morbidity due to hypertensive disorders (RR = 0.80, 95% CI 0.65–0.97), anti-microbial prophylaxis in reducing the odds of puerperal sepsis/postpartum endometritis (OR = 0.49, 95% CI 0.23–1.06), anti-malarial prophylaxis in reducing the risk of severe antenatal anemia (RR = 0.62, 95% CI 0.50–0.78), and iron supplementation in reducing the risk of iron deficiency anemia at term (RR = 0.33, 95% CI 0.16–0.69).

**Conclusion:**

Maternal mortality could be reduced by a combination of micronutrient supplementation and presumptive treatment of infection during pregnancy. Such an approach could be adopted in resource-poor settings where visits to antenatal clinics are infrequent and would complement existing Safe Motherhood activities.

## Background

The Millennium Development Goal of halving maternal mortality by 2015 is unlikely to be achieved in sub-Saharan Africa, where little or no progress has been reported [1]. Current strategies to save the lives of pregnant women rightly focus on skilled birth attendance or emergency obstetric care [2]. There is no doubt that these strategies could save many lives. However, it is also clear that such strategies require health care professionals that the poorest countries cannot afford to train or retain in sufficient numbers over the next decade [3]. More importantly, these strategies might be insufficient in view of the increasing proportion of maternal deaths attributable to infectious disease, particularly in sub-Saharan Africa [4].

The majority of women in developing countries come into contact with health care workers during pregnancy through antenatal care [5]. Antenatal care for maternal health has been marginalised for the last 15 years because of poor risk scoring predictability [2]. The effectiveness of various components of antenatal care has been studied, but the overall potential impact on maternal mortality has not been estimated, as it has been for neonatal outcomes [6,7].

In this paper, we ask whether a simple antenatal package which combines micronutritional supplementation with presumptive treatment of infection, given through antenatal care or taken at home under limited supervision, could prevent some of the 250,000 maternal deaths which have been estimated to occur each year in sub-Saharan Africa [8]. Our objective was to estimate the impact of such a package on maternal mortality in sub-Saharan Africa, based on the distribution of the causes of maternal deaths in the region, and based on evidence for the effectiveness of each component. Our broader aim was to demonstrate the potential importance of antenatal interventions in developing countries, and to help define a simple package which could be provided to women who access existing antenatal care, but which could also be delivered via outreach programmes to the 25% of women who fail to make even one antenatal visit.

## Methods

Our study is a model-based analysis, which uses a recent WHO systematic review of causes of maternal deaths [9]. We also used observational studies, reviews, and baseline data from controlled trials to estimate the prevalence among pregnant women in sub-Saharan Africa of aetiological factors such as micronutrient deficiency and infection associated with reported causes of maternal deaths.

We obtained evidence for the effectiveness of antenatal interventions with the potential to prevent maternal deaths due to severe hypertensive disorders, sepsis, malaria, and iron-deficiency anemia from The Cochrane Library [10].

We first calculated the crude reduction in all-cause maternal mortality which could be achieved through full (100%) population coverage of the intervention, compared with existing coverage, by multiplying the efficacy of the intervention by the proportion of all-cause maternal deaths attributable to the preventable cause. Hence, the percentage reduction *r *in maternal mortality due to an intervention against a particular cause of death is given by Equation 1.

*r *= (1 - *RR*) × *p*

where *RR *= risk or odds ratio for cause of death comparing intervention with no intervention, and *p *= proportion of all-cause maternal mortality due to a particular cause of death. For each cause of death we calculated a range for the percentage reduction *r *by substituting into the equation the upper and lower bounds of the 95% confidence interval for *RR *and the upper and lower bounds of the range of *p*.

To obtain a point estimate and range for the total reduction in all-cause maternal mortality, we used a model with two stages. In the first stage, we re-created the distribution of *RR *for the reduction in maternal mortality due to the intervention for each of cause of death by calculating the error factor for each *RR *estimate from the 95% confidence interval (Equation 2, [11]), and taking the mean of the error factor estimates calculated using the upper and lower 95% confidence interval. A normal distribution using the natural log of *RR *as the mean, and the standard error of log(*RR*) (calculated using the error factor, Equation 3, [11]) as the variance, was created for each cause of death (using Berkeley Madonna version 8.3.11, University of California at Berkeley), and 50,000 values of log(*RR*) were selected at random from this distribution and transformed to give 50,000 estimates of *RR*. Equation 1 was used to calculate *r *for each *RR *estimate.

95%*CI*(*RR*) = *RR*/*EF *to *RR *× *EF*

*EF *= exp [1.96 × *SE*(log *RR*)]

A variable proportion of maternal deaths caused by anemia are attributed to malaria; we selected at random from a uniform distribution for this proportion, with lower bound 0% (none of the anemia deaths attributed to P *falciparum *infection, representing the few parts of sub-Saharan Africa free of malaria), and upper bound 18% (from an estimate by Brabin et al, representing holoendemic areas, [12]). This variation was incorporated into the first stage of the model. Of the remaining deaths caused by anemia, a variable proportion are attributed to iron deficiency. Our review of the literature suggested a range of 30–70% for this proportion [12-18]. We incorporated the lowest, middle and highest point of this range into the second stage of our model, as described in the next paragraph.

In the second stage, we obtained the overall percentage reduction in all maternal deaths estimated for the intervention. For each of the 50,000 values of *RR *that we generated for the effect on each cause of death, we subtracted the proportion of deaths prevented due to the intervention for one cause of death before calculating the effect on the next cause of death; this method takes into account independent co-morbidity, and does not depend on the order in which causes of death are chosen [7]. The proportions of deaths prevented at the 2.5^th ^and 97.5^th ^percentile were used as 95% uncertainty intervals. These calculations were repeated using the point estimate and the lowest and highest points of the range of the proportion *p *of maternal deaths due to each cause found by the recent WHO systematic review of causes of maternal deaths [9] to produce three scenarios. At the lowest end of the range of *p*, we used the lowest proportion (30%) of non-malarial anemia deaths attributed to iron deficiency; at the highest end of the range of *p*, we used the highest proportion (70%) of non-malarial anemia deaths attributed to iron deficiency.

## Results

We identified severe hypertensive disorders, puerperal sepsis and anemia as causes of maternal deaths which could be prevented by micronutrient supplementation and presumptive treatment of infection during the antenatal period. Current evidence for the effectiveness of antenatal interventions for preventing maternal deaths due to these causes is summarised in Table 1. We could not identify any simple prophylactic or nutritional interventions which have potential to prevent the other causes of maternal deaths identified by the WHO systematic review (haemorrhage, obstructed labour, abortion, HIV/AIDS).

**Table 1:** Causes of maternal deaths and evidence for preventive antenatal interventions.

Cause of death	Aetiology	Antenatal intervention	Evidence base	Grade*
Severe hypertensive disorders (eclampsia and pre-eclampsia)	Dietary calcium deficiency	Calcium supplementation	"Calcium supplementation appears to almost halve the risk of pre-eclampsia, and to reduce the rare occurrence of the composite outcome 'death or serious morbidity"' [19].	High
Puerperal Sepsis	Genitourinary infection	Antibiotics	"Antibiotic prophylaxis given during the second or third trimester of pregnancy reduces the risk of pre-labour rupture of membranes when given routinely to pregnant women. Beneficial effects on birth weight and the risk of postpartum endometritis were seen for high-risk women" [22].	Moderate
	Dietary vitamin A deficiency	Vitamin A supplementation	"Further evidence on biologically plausible mechanisms of morbidity reduction such as reduced incidence of sepsis and/or reduced markers of inflammation are needed" [60].	Low
	Dietary micronutrient deficiencies	Multiple micronutrient supplementation	One trial in Tanzania showed no additional effect on markers of infection (C-reactive protein) compared with iron and folic acid [15].	Low

Anemia	*P. falciparum *infection	Anti-malarial drugs	"Drugs given routinely for malaria during pregnancy reduce severe antenatal anaemia in the mother, and are associated with higher birth weight in the baby and probably fewer perinatal deaths. This effect appears to be limited to low parity women" [24].	High
	Dietary iron deficiency	Iron supplementation	"Antenatal supplementation with iron or with iron and folic acid results in a substantial reduction in the prevalence of haemoglobin levels below 10 or 10.5 g/L at term or near term. Routine daily or weekly antenatal iron or iron plus folic acid supplementation may be of benefit, especially where pre-gestational iron deficiency and anaemia are prevalent" [26].	Moderate
	Dietary vitamin A deficiency	Vitamin A supplementation	"Further evidence on the effectiveness of adding vitamin A to iron and folic acid for treatment of anaemia is needed" [60]. "Vitamin A supplementation programmes to reduce anaemia should not be implemented in similar antenatal populations in rural sub-Saharan Africa unless evidence emerges of positive benefit on substantive clinical outcomes" [61].	Low
	Dietary micronutrient deficiencies	Multiple micronutrient supplementation	When compared with supplementation of two or less micronutrients or no supplementation or a placebo, multiple-micronutrient supplementation resulted in a statistically significant decrease in maternal anaemia (RR 0.61; CI 0.52 to 0.71). However, these differences lost statistical significance when multiple-micronutrient supplementation was compared with iron folic acid supplementation alone [64].	Low
	Intestinal parasitic infection	Anthelmintic/antiprotozoal drugs	Trials in Sierra Leone and Nepal showed a positive effect on haemoglobin levels [57,59].	Moderate

* GRADE Working Group system [65]

### Severe hypertensive disorders

A protective effect of calcium supplementation against severe hypertensive disorders (pre-eclampsia and eclampsia) has been demonstrated among women who have insufficient dietary intake of calcium [19]. We found two studies which reported on dietary calcium intake among pregnant women in sub-Saharan Africa, and both studies reported average intakes substantially below the recommended daily allowance for pregnant women of 1200 mg/day [20,21]. In the absence of further studies, we assumed that low calcium intake (<600 mg/day) was widespread across sub-Saharan Africa. We applied the 20% (1-*RR *= 0.03–0.35) reduction in risk of maternal death/serious morbidity reported by the Cochrane Review (RR = 0.80, 95% CI 0.65–0.97), corresponding to this level of calcium intake, to the 3.9–21.9% of maternal deaths attributed to severe hypertensive disorders, to obtain a reduction in maternal mortality of 0.1–7.7%, corresponding to 300–19,200 deaths per year (Table 2).

**Table 2:** Estimated crude reduction in maternal mortality due to micronutritional supplementation and presumptive treatment of infection, by cause of maternal death.

Cause of maternal death	Proportion of all maternal deaths (range)^1^	Intervention	Effectiveness of intervention – reduction in deaths by cause (95% CI)^2^	Crude reduction in all-cause maternal mortality^3^	Maternal deaths prevented per year^4^
Severe hypertensive disorders	9.1% (3.9–21.9)	calcium supplementation	20% (3–35)	0.1–7.7%	300–19,200
Puerperal sepsis	9.7% (6.3–12.6)	anti-microbial prophylaxis	51% (0–77)	0.0–9.7%	0–24,300
Anemia	3.7% (0.0–13.2)	anti-malarial prophylaxis	38% (22–50)	0.0–1.2%^5^	0–3,000
		iron supplementation	67% (31–84)	0.0–6.4%^6^	0–15,900

^1 ^Source: WHO systematic review of causes of maternal deaths [9].

^2 ^Source: Cochrane Reviews [19,22,24,26].

^3 ^Calculated using Equation 1.

^4 ^Assuming 250,000 deaths per year in sub-Saharan Africa [8].

^5 ^Assuming 18% of maternal deaths caused by anemia are attributable to P *falciparum *infection [12].

^6 ^Assuming 30–70% of non-malarial maternal deaths caused by anemia are attributable to iron deficiency [12-17].

### Puerperal sepsis

The Cochrane Review of anti-microbial prophylaxis during pregnancy (2^nd ^and 3^rd ^trimester) gives an odds ratio for puerperal sepsis/postpartum endometritis among women selected for poor obstetric history (low birth weight or stillbirth) of 0.46 (95% CI 0.24–0.89); this result is based on a single trial in Kenya [22]. Among unselected women the odds ratio is not quite significant (OR = 0.49, 95% CI 0.23–1.06). The result for unselected women is based on two trials, one conducted in the USA (OR = 0.68, 95% CI 0.23–2.00) and one conducted in Kenya (OR = 0.36, 95% CI 0.12–1.06). Applying the 51% risk reduction (1-*RR *= 0.00–0.77) reported in the Cochrane Review for unselected women to the 6.3–12.6% of maternal deaths attributed to puerperal sepsis (assuming no adverse effect of the intervention) yields a reduction in maternal mortality of 0.0–9.7%, corresponding to 0–24,300 deaths per year (Table 2).

### Anemia

In our crude calculations, we used the estimate by Brabin et al that, in malarious areas, malaria would be the underlying cause of 18% of maternal deaths due to anemia (hence 0–2.4% of all maternal deaths) [12]. The remainder (0–10.8% of all maternal deaths) would be caused by nutritional deficiencies (mainly iron deficiency), sickle cell disease, and infection (mainly intestinal parasites such as hookworm). Studies suggest that 30–70% of all maternal anemia is due to iron deficiency [12-17]; hence iron deficiency would be the underlying cause of 0–7.6% of all maternal deaths.

### Anemia due to malaria

WHO recommends preventive intermittent treatment for *falciparum *malaria [23]. The Cochrane Review of anti-malarials during pregnancy gives a relative risk for severe antenatal anemia during first and second pregnancies of 0.62 (95% CI 0.50–0.78) [24]. Applying this 38% risk reduction (1-*RR *= 0.22–0.50) to the 0–2.4% of maternal deaths caused by malaria (assuming that the majority of deaths occurred among primi- and secundigravid women) gives a reduction in maternal mortality of 0–1.2% corresponding to 0–3,000 deaths per year (Table 2).

### Anemia due to iron deficiency

WHO recommends routine iron supplementation during pregnancy [25]. The Cochrane Review of iron supplementation with or without folic acid during pregnancy gives a risk ratio for iron deficiency anemia at term (Hb below 110 g/L and at least one additional laboratory indicator) of 0.33 (95% CI 0.16–0.69) [26]. Applying this 67% risk reduction (1-*RR *= 0.31–0.84) to the 0–7.6% of maternal deaths caused by severe iron deficiency anemia gives a reduction in maternal mortality of 0–6.4% corresponding to 0–15,900 deaths per year (Table 2).

### All causes

Using a range of 0–18% for the proportion of anemia due to malaria and a range of 30–70% for the proportion of non-malarial anemia due to iron deficiency in our scenario analysis, our model generated distributions for the overall reduction in maternal deaths with median values 3.9%, 7.7% and 15.6%, and 95% uncertainty intervals of 0.4–5.7%, 2.4–10.7%, and 8.3–20.3% for the lower bound, point estimate and upper bound respectively of the proportion of maternal deaths due to each cause from the WHO systematic review (Table 3). Hence our overall estimate for the proportion of maternal deaths prevented is 8%, with a range of <1–20%, corresponding to 19,300 (range 900–50,800) maternal deaths prevented per year in sub-Saharan Africa.

**Table 3:** Estimated reduction in all-cause maternal mortality due to micronutritional supplementation and presumptive treatment of infection.

	Lowest estimate	Point estimate	Highest estimate
Proportion of maternal mortality due to^1^:			
Pre-eclampsia/eclampsia	3.9	9.1	21.9
Puerperal sepsis	6.3	9.7	12.6
Anaemia (all causes)	0.0	3.7	13.2
Proportion of non-malarial anaemia due to iron deficiency^2^	30%	50%	70%
Percentage reduction in all-cause maternal mortality, median (uncertainty interval)	3.9% (0.4–5.7)	7.7% (2.4–10.7)	15.6% (8.3–20.3)
Number of maternal deaths prevented per year, median (uncertainty interval)	9,800 (900–14,200)	19,300 (6000–26,600)	38,900 (20,700–50,800)

^1 ^Source: WHO systematic review of causes of maternal deaths [9]

^2 ^Source: references [12-17].

## Discussion

Our study suggests that a substantial number of maternal deaths could be prevented in sub-Saharan Africa by nutritional and anti-infection interventions in pregnancy. The range of our estimate derives from uncertainty about the causes of maternal deaths, and uncertainty about the effectiveness of antenatal interventions. We question why there is so little emphasis on the potential of these antenatal strategies by the international safe motherhood community, and why the initiative has been left to vertical programmes, such as malaria treatment and prevention. Of course, we recognize that there is no 'silver bullet' strategy for addressing the effects of a lifetime of undernutrition and infection on maternal health. However, we believe that any evidence-based intervention which could be made available to women within the constraints of existing health infrastructure or through a community-based outreach approach merits further exploration.

### Limitations

Evidence of effectiveness based on large-scale randomised controlled trials of unselected or unscreened population groups in low-income countries was lacking for presumptive treatment of genitourinary infections and micronutrient supplements, with the exception of calcium. Evidence of effectiveness in preventing maternal mortality was lacking for all interventions. Instead we have assumed that prevention of mortality is in direct proportion to reduced risk of morbidity. This assumption is more reasonable for calcium supplementation and anti-malarial prophylaxis, where there is evidence of effectiveness in preventing severe morbidity, than for iron supplementation and anti-microbial prophylaxis, where the evidence of effectiveness relates to less severe morbidity.

We have taken into account independent comorbidity between conditions, eg. anemia and infection, malaria and pre-eclampsia, but not dependent comorbidity, nor possible interaction between the components, eg. between iron and calcium, between iron and vitamin A, and between *P falciparum *and micronutrient deficiencies [13,27,28]. We have produced an estimate for sub-Saharan Africa, but the region is homogeneous neither in the causes of maternal mortality nor in the prevalence of micronutrient deficiencies and infection. The WHO systematic review of causes of maternal deaths included one study from Egypt, but all of the other included studies were conducted in countries south of the Sahara.

Our estimate of the impact of the combined intervention is a best-case scenario, based on 100% coverage compared with whatever coverage existed in the areas where the mortality studies were conducted. Although it is estimated that 68% of women across sub-Saharan Africa have at least one antenatal consultation [5], the actual provision and use of treatment and prophylaxis at these consultations is largely unknown, and is unlikely to be homogeneous across sub-Saharan Africa. Even if full coverage were to be achieved among women who currently access antenatal care, the impact of the intervention would vary across the region, and would also be influenced by adherence to supplementation regimens.

We decided that another layer of estimates (of current and projected coverage) and corresponding calculations would over-complicate our model. However, it is a fairly straightforward exercise to calculate the factor by which the impact of each intervention would be reduced for less than full coverage. For example, if two-thirds of women who currently access antenatal care receive iron prophylaxis and anti-malarials (hence 45% of all women), and full coverage is somehow achieved among women who currently access antenatal care (68% of all women), then the impact of these two interventions on deaths due to anemia will be reduced by a factor of 0.4 (68-45/100-45). The impact of the two new interventions (calcium supplementation and presumptive treatment of genitourinary infection) will be reduced by a factor of 0.7 (68/100). The upper bound of our uncertainty interval for the reduction in maternal deaths will be around 15%.

### Severe hypertensive disorders

The Cochrane Review estimate of the effect of calcium supplementation in reducing maternal mortality due to severe hypertensive disorders is based almost entirely on the result of a WHO multi-centre trial conducted among women with low dietary calcium intake [29]. We have made the assumption that diets across sub-Saharan Africa are unlikely to provide the 1200 mg recommended daily allowance for pregnant women. Mechanisms to ensure adherence may become an important issue given the physical size of calcium tablets. This issue is further complicated by the inhibition of iron absorption by calcium hence there is likely to be a trade-off between the benefits of each supplement. The WHO trial used three chewable 500 mg tablets, one tablet to be taken three times daily at meals but three hours away from any iron supplements [29]. However, in settings where management of pre-eclampsia/eclampsia is difficult due to lack of resources, a preventive strategy might be a worthwhile weapon against maternal deaths due to hypertensive diseases of pregnancy.

Studies from Senegal and The Gambia have reported an increased risk of death due to eclampsia, RR = 3.1 (95% CI 0.8–14.7) and RR = 5.4 (95% CI 1.5–19.3) respectively, coincident with peak transmission of P *falciparum *[30,31]. A three-fold increase in the risk of pre-eclampsia in women presenting with placental parasitemia was also reported from a hospital-based study in Dakar [32]. Hence treatment of malaria might be expected to prevent some deaths due to pre-eclampsia/eclampsia [33]. Supplementation with vitamins C and E has been shown to have no effect on eclampsia, albeit among well-nourished women [34,35].

### Puerperal sepsis

The inclusion of presumptive treatment of genitourinary infection, and its estimated impact on puerperal sepsis, is likely to raise concerns about resistance to antibiotics. The data from Kenya strongly suggested a beneficial effect of mass anti-microbial treatment in pregnancy where sexually transmitted infections are highly prevalent [36]. In the fourteen years since this trial was conducted, new antibiotics have become available [37]. Azithromycin, for example, has demonstrated efficacy against chlamydia, gonorrhoea, chancroid, incubating syphilis, mycoplasma, and possibly bacterial vaginosis, and appears to be without teratogenic risk [38-44]. Azithromycin has been used for mass treatment of other infections, and has demonstrated efficacy against *P falciparum *and *P vivax *[45-47]. Three trials conducted since the Cochrane review have provided further evidence, at least for perinatal outcomes, of the benefits of prophylactic antibiotics in pregnancy [48].

WHO currently recommends a range of anti-microbials for treatment of genitourinary infections during pregnancy, some prescribed over several days [25]. It is widely acknowledged that current strategies for treating genitourinary infection among pregnant women in sub-Saharan Africa are failing, and that new approaches are needed [49-52]. A successful strategy could also contribute to the prevention of HIV transmission [53]. However, because of the likely controversy, we would advocate for a large-scale trial to establish the efficacy and safety of this strategy in settings where the prevalence of sexually transmitted infections is high.

### Anemia

We have based our estimate on the effects of anti-malarial treatment and iron supplementation. Although the benefits of intermittent presumptive treatment (IPT) are greatest among women of low parity, it has been suggested that IPT should be provided to all pregnant women in areas of high HIV prevalence because HIV-positive multigravidae are at similar risk of severe malaria as HIV-positive primigravidae [54].

Nutritional deficiencies are compounded by other infections, principally hookworm [55-57]. Pathogens such as Helicobacter pylori may also play a major role [58]. Some of the maternal deaths caused by anemia, which were attributable neither to malaria nor to iron deficiency, might be prevented through a combination of multiple micronutrient supplementation and presumptive treatment of parasitic and bacterial infection [55,59]. Albendazole has been shown to reduce the decline in haemoglobin during pregnancy, but there is no conclusive evidence for an effect of vitamin A or other micronutrients on anemia during pregnancy, particularly when compared with iron and folate supplementation [60,61].

### Other benefits

Each component of the combined intervention would benefit neonatal health and survival [7]. Presumptive treatment of sexually transmitted infections would act directly to prevent perinatal deaths due to syphilis and sepsis; treatment and prevention of maternal anemia, malaria and pre-eclampsia/eclampsia would act indirectly via higher birth weights and fewer preterm births.

### Delivery and cost-effectiveness

The interventions could be combined in a package designed to facilitate distribution by health providers and to maximise adherence by patients. Delivery of such a package need not be confined to health facilities and could be distributed by community health workers and others involved in community outreach programmes. Ideally, the package would be implemented in conjunction with measures to improve access to family planning, skilled attendance and essential obstetric care.

Studies of the cost-effectiveness of several of the interventions, particularly with regard to neonatal outcomes, suggest that a combined intervention is likely to be cost-effective in averting maternal and neonatal mortality and morbidity [7,62,63]. However, considerable gaps in our knowledge remain, particularly with regard to cost-effectiveness of micronutritional supplementation, and further research is required. Such research would need to consider targeted delivery to high-risk groups such as primiparous women, screening as an alternative to presumptive treatment, and the relative cost-effectiveness of other maternal health interventions such as provision of essential obstetric care.

### Recommendations

Our study is a type of sensitivity analysis, based on the available evidence. Although the point estimates for the effectiveness of the constituent interventions could be debated, the strength of evidence for each manifests as the upper and lower bounds of the 95% confidence intervals. These uncertainties combine with the ranges for the causes of death and underlying prevalence of micronutrient deficiencies and infection, to generate the uncertainty interval for our final estimate (<1% to 20%). The lower and upper bounds of this interval almost certainly under- and over-estimate the impact of combined nutritional and anti-infection interventions during pregnancy. A narrower interval could be obtained through the gradual accumulation of evidence from studies and trials, and perhaps a more sophisticated model. However, we would argue that there is sufficient evidence for each intervention to support a large cluster randomised controlled trial of a package combining these interventions, compared with implementation of current WHO guidelines for antenatal care. The intervention and comparison groups could each have two arms: one based on the prevalent (presumably facility-based) model of antenatal care; the other providing an enhanced model of antenatal care comprising facility-based and community outreach teams.

## Conclusion

Our estimate for the reduction in the maternal mortality ratio which could be achieved through a package combining routine multiple micronutrient supplementation with presumptive treatment of parasitic and bacterial infection is 8% (range <1–20%). Such a package would complement the Safe Motherhood measures of skilled birth attendance, emergency obstetric care and family planning. We trust that the model which we have presented will serve to inform the debate over prioritisation of resources and to highlight the number of maternal deaths which could be prevented through the relatively simple measure of implementing and building upon existing antenatal care recommendations.

## Competing interests

The author(s) declare that they have no competing interests.

## Authors' contributions

VF and RP raised the research question. SC and RB conducted the research and calculated the estimates. VF and RP provided programmatic and clinical judgements. SC wrote and revised the drafts, RB designed and ran the models; VF, RP and RB provided input at each revision.

## Pre-publication history

The pre-publication history for this paper can be accessed here:


